# What is needed now for successful scale-up of screening?

**DOI:** 10.1016/j.pvr.2019.04.011

**Published:** 2019-04-16

**Authors:** Silvia de Sanjose, Francesca Holme

**Affiliations:** PATH, Seattle, USA

**Keywords:** HPV, Cervical cancer, Screening, Follow-up, Barriers, Health information system

## Abstract

Effective screening for pre-cancerous lesions of the cervix is the only protective intervention that can be offered to women that have not had the opportunity to be vaccinated. Elimination goals are being developed so that by 2030, 70% of women aged 35–45 years should have been screened at least once in a lifetime and 90% of all detected lesions should have been treated. These goals focus on a substantial reduction of cervical cancer burden in low- and middle-income countries (LMICs). Scaling-up screening in these settings may be substantially improved by using self-sampling (SS), human papillomavirus (HPV) testing, and managing screened-positive women with accessible treatment. The implementation of these tools requires minimal health information data for traceability, provider training, community education, operational management and quality control. Cost-effective algorithms tailored to country needs can greatly impact the burden of disease in a limited number of years.

## 1 Screening as an opportunity to save lives

Cervical cancer incidence and mortality remain important indicators of global health inequality. Today, despite being a largely preventable disease, cervical cancer is the fourth most common cancer among women globally. With an estimated 569,000 new cases and 313,365 new deaths in 2018, nearly 90% of these deaths were in LMICs^1^ [1].

In May 2018, the WHO^2^ Director General made a global call for action towards the elimination of cervical cancer [2] based on proven strategies across the care continuum. Strategies to reach elimination include vaccination to prevent HPV^3^ infection, screening and treatment of pre-cancerous lesions, and early detection and prompt treatment of early invasive cancers. At the time of this writing, specific elimination targets are still under public consultation, and include vaccination of 90% of girls below age 15, screening of 70% of women aged 35–45 years at least once in a lifetime and treatment of 90% all women with detected lesions. While the pace and timeline of success will vary across the globe, it is expected that a major decrease in the burden will be attainable in the next decade.

**Why screen?** Although vaccination will have a major impact on disease reduction [3], the large majority of women living in LMICs have not had the opportunity to be vaccinated [4]. Effective screening for pre-cancerous lesions of the cervix is the only protective intervention that can be offered to these women [5]. Otherwise, the women alive today who have not benefitted from vaccination will generate about 35–40 million cancer cases over the next 65 years [6].

**Who to screen?** When investing in scaling-up cervical cancer screening, a critical issue is the ability to provide optimal management of screen-detected lesions. Historically, this has been one of the major challenges in LMICs [5]. Management of 90% of all screen-detected lesions is an extremely ambitious aim, particularly in countries with limited technical capacity, infrastructure, and management facilities. Prior to embarking on an effort to target the population to be screened, it is important to assess the health system's capacity to manage screen-detected pre-neoplastic lesions and cancer cases, and build additional capacity where needed.

## 2 Scaling-up in LMIC

There are several strategies described in Table 1 that can effectively facilitate scaling-up screening programs. Scaling up screening of cervical cancer will require an organized approach within health care systems that is tailored to the needs of the country, in order to achieve high coverage of population-level screening and cost-efficient use of resources [7]. Each country or screening catchment area needs to develop accurate, detailed, and clear guidelines of the screening process, defining target ages, interval between tests based on screening results, triage tests, follow up, and referral indications. Guidelines must be accompanied by operational instructions that allow providers to understand how to implement the procedures, and must be approved by relevant authorities in the country. Guidelines and operational protocols should provide a full consideration of the following aspects:

a) **Primary screening test:** Critical issues include sample method and test. Implementing self-sampling^4^ of vaginal cells allows accurate detection of HPV infection and provides an opportunity to maximize coverage in low-resource settings compared to provider-collected samples of cervical cells [8,9]. SS is a highly cost-effective approach for health systems, as gynecological exams will be required only for women who screen positive or for those reluctant to SS [10]. Women's acceptance of SS in LMICs is high. Additionally, it is critical to select an **HPV assay** that is validated, affordable, and easy to run. WHO has pre-qualified two HPV assays for introduction in LMICs, GeneXpert and *care*HPV. Although both assays are being used in LMICs, they vary in sensitivity, management, and price. Thus, the selection will largely depend on affordability. Additional assays specifically designed for LMIC markets are currently under development. The use of Pap test is not recommended.
b) **Managing screen-positives:** A single-visit approach is highly recommended, minimizing loss to follow-up for triage and treatment. Once the HPV test result is known, screen-positives undergo triage or proceed to immediate treatment. In LMICs, VIA^5^ is a common triage strategy used either to identify large or cancerous lesions to send for referral, and treat all remaining HPV positives, or to identify eligible lesions to be treated with thermal ablation, leaving HPV-positive VIA-negative women for follow up at a later date [7]. The use of Pap as a triage test is not recommended as it adds complexity and delays managing patients [7]. Recent data suggest that using EVA^6^ together with the AVE^7^ system on the images could increase the quality of the triage step substantially at an affordable level [11]. Other triage tests such as methylation assays, dual staining, and E6/E7 oncogene detection are promising but need to be tested for high throughput in low-resource settings [12]. Their advantage is that they could use the same biological sample that is being used for the HPV test, thus minimizing additional clinic visits.

**Table 1:** Critical aspects that may favor scaling-up screening activities by level of the intervention.

Level	Step	Test	Favor scaling-up
Primary screening	Sampling	Self-sampling	+++
		Provider-collected sample	+
	Test	HPV: accurate, acceptable, automated^a^	+++
		VIA	+
		Pap	–
Managing screen positive	Triage	VIA for treatment eligibility only	++
		VIA (+/− enhanced visualization)	++
		EVA + VIA	Evidence in progress
		Molecular tests^a^	Evidence in progress
		Pap	–
	Treatment precancer	Thermal ablation	+++
		Cryotherapy	++
		LLETZ	+
Traceability^b^	Screen positive	Minimal data required	+++
	Triage positive	Minimal data required	+++
	Treated patients	Minimal data required	++
Provider training and education	At all steps & community		+++
Quality control	At all levels		+

HPV: human papillomavirus; VIA: visual inspection with acetic acid; EVA: enhanced visual assessment; LLETZ: large loop excision of the transformation zone.

a Important variations in price exist.

b Traceability must be embedded in scaling up to guaranteed adequate management and avoiding over-screening.

Treatment of pre-cancerous lesions and referral of small, visible acetowhite lesions can be managed at the primary health care level using thermal ablation or cryotherapy, with similar efficacy. The need for cryogas is the main bottleneck in delivering cryotherapy treatment, making thermal ablation a more attractive alternative. Lesions covering more than 75% of the transformation zone, affecting the canal, or suspicious for cancer need to be referred to advanced care facilities for additional management. In the latter cases, it is of utmost importance that women are guided through the system to guarantee that treatment is available and that financial cost is not impeding the process.

**Fig. 1:**
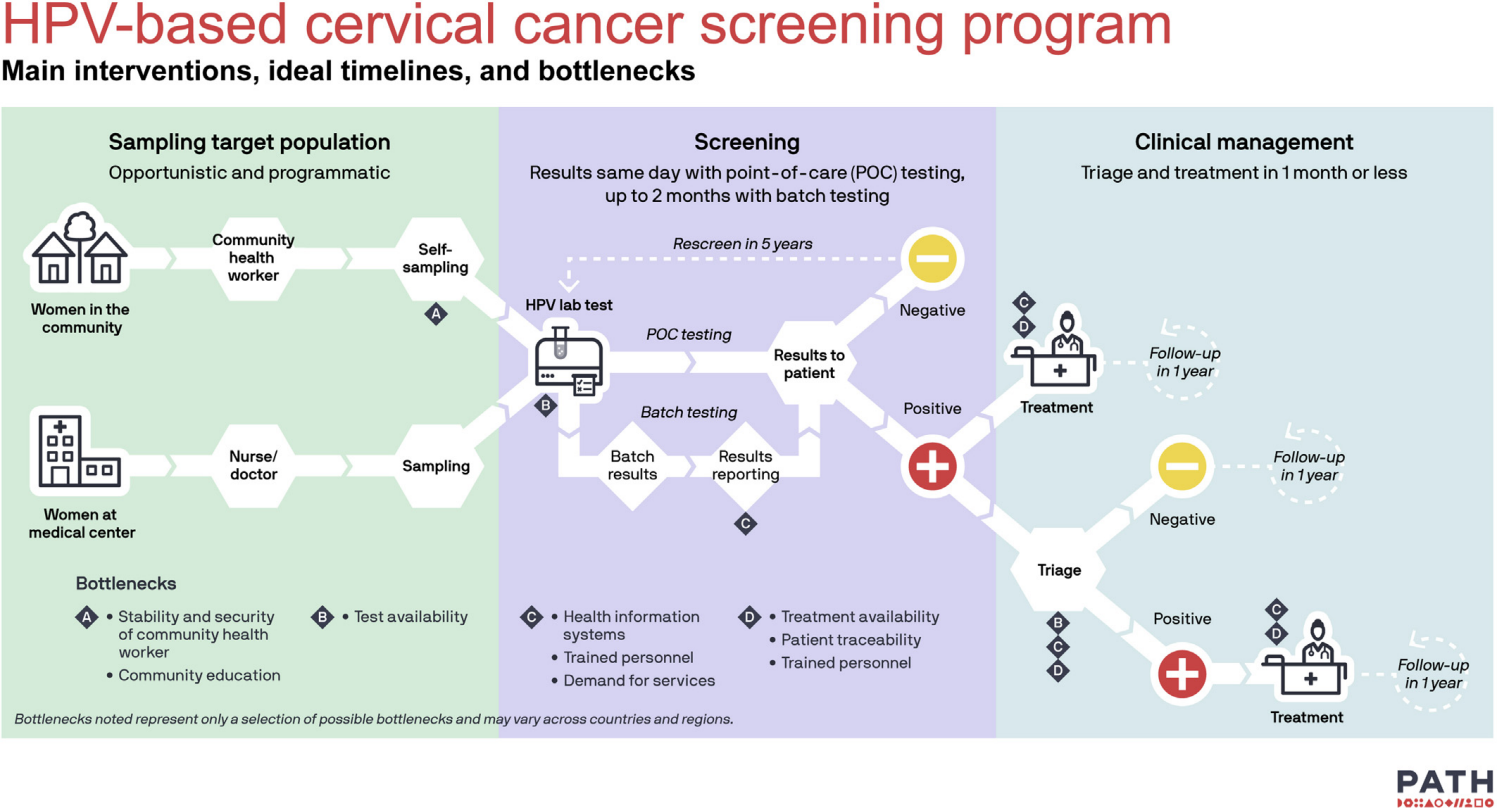
Generic algorithm of a HPV-based cervical cancer screening program including main interventions, ideal timelines and main bottlenecks.

Fig. 1 outlines the screening process in three key areas: the taking of the sample, HPV testing, and clinical management of screen-positives. The figure depicts the theoretical timelines and main bottlenecks observed in some settings, aiming to illustrate that screening does not end at a sample being taken or at lab testing. Screening implies management and follow-up of screen-positives.

c) **Traceability:** Health information systems must allow health care providers to track screened women, thus enabling follow-up and treatment completion. At all levels of the health care system, generating a basic set of indicators on coverage, treatment access, quality, and detected lesions will provide essential feedback that enables providers to measure performance and maximize efficiency. The inclusion of a basic minimal number of information elements against extensive record data will likely increase completeness.
d) **Training and education**: Changing routines can be cumbersome and may generate resistance at many levels of the healthcare system. Training providers and carrying out community education and outreach activities are critical for generating an adequate demand, a good flow of patients into the program, and adherence to guidelines. Educational material and flowcharts are helpful elements to be created for a wide participation of stakeholders.
e) **Quality control:** Establishing key quality-control measures to guarantee that the activities performed are in line with the screening objectives is advisable. It may not modify the scaling-up of the process but may facilitate the identification of inadequate steps, poor training, or malpractice.

Screening for cervical cancer aims to protect women from developing cervical cancer. Scaling-up screening in LMICs may be substantially improved through self-sampling approaches, HPV testing, improved triage and managing screen-positive women with accessible treatment[13]. The implementation of these tools requires basic minimal health information for traceability, provider training, community education, operational management, and quality control. Algorithms tailored to individual country needs can greatly and positively affect the impact on burden of disease in a limited number of years.

Ultimately, the effectiveness and impact of the screening process will have to be measured through incidence parameters obtained from cancer registries and/or disease-related mortality.

## Conflict of interest

None.

## Footnotes

1 LMICs: low- and middle-income countries.

2 WHO: World Health Organization.

3 HPV: human papillomavirus.

## Footnotes

4 SS: self-sampling.

5 VIA: Visual inspection with acetic acid.

6 EVA: enhanced visual assessment.

7 AVE: automated visual evaluation.
